# High incidence of type III secretion system associated virulence factors (exoenzymes) in *Pseudomonas aeruginosa* isolated from Iranian burn patients

**DOI:** 10.1186/s13104-019-4071-0

**Published:** 2019-01-15

**Authors:** Ramin Khodayary, Iraj Nikokar, Mohammad Reza Mobayen, Farhad Afrasiabi, Afshin Araghian, Ali Elmi, Meisam Moradzadeh

**Affiliations:** 1 Department of Microbiology, Faculty of Medicine, Guilan University of Medical Sciences, Rasht, Iran; 2 Medical Biotechnology Research Center, School of Paramedicine, Guilan University of Medical Sciences, Rasht, Iran; 3 Department of Surgery, Guilan University of Medical Science, Rasht, Iran; 4 Laboratory of Microbiology and Immunology of Infectious Diseases, Paramedicine Faculty, Guilan University of Medical Sciences, P.O. Box: 44715-1361, Langeroud, IR Iran

**Keywords:** *Pseudomonas aeruginosa*, Virulence factors, Antimicrobial resistance, Burn patients, Type III secretion system

## Abstract

**Objective:**

The present study aimed to determine the prevalence of virulence factors and antimicrobial resistance profile of *Pseudomonas aeruginosa* strains isolated from Iranian burn patients.

**Results:**

This cross-sectional study performed on 100 *P. aeruginosa* isolates which were recovered from burn wound specimens in 2014–2015. All presumptive isolates were identified by standard microbiologic tests. Antimicrobial susceptibility test was carried out by disk diffusion method. The presence of virulence genes was determined by PCR method. Antibiotic susceptibility results revealed that the isolates were mostly susceptible to amikacin (61%), ceftazidime (60%), and imipenem (55%). Moreover, 59% of the isolates were multi-drug resistance (MDR). The most prevalent MDR pattern was aminoglycosides–penicillins–fluoroquinolones–carbapenems (15%). The presence of *exoT*, *exoY*, *exoS* and *exoU* genes was detected in 100%, 100%, 59%, and 41% of the tested isolates, respectively. Results points out the pattern of MDR and genetic diversity of type III secretion system among *P. aeruginosa* strains isolated from the burn population. Overall, the association of MDR and the presence of the specific virulence genes can be a predictive marker for the persistence of these isolates in the hospitals and subsequently a worse clinical condition for the affected patients.

## Introduction

*Pseudomonas aeruginosa* is an opportunistic Gram-negative pathogen that has been considered as a major cause of nosocomial infections, particularly in immunocompromised or patients with underlying diseases such as burn wounds [1, 2]. *P. aeruginosa* causes a variety of acute infections in hospitalized patients associated with a high rate of morbidity and mortality [3]. Environmental contamination and direct spread from patients or healthcare workers are the most frequent reservoirs of *P. aeruginosa* in healthcare settings [4].

Pathogenicity of *P. aeruginosa* is attributed to the production of several extracellular and cell-associated virulence factors including those implicated in adherence, iron uptake, exoenzymes and exotoxins [5, 6]. Among the diversity of virulence determinants, the type III secretion system (T3SS) is considered as an important factor resulting in poor clinical outcome of *P. aeruginosa* infections in burn patients [7, 8]. To date, this system is believed to be responsible for the injection of at least 4 effector proteins in *P. aeruginosa*, including Exoenzyme S, Exoenzyme T, Exoenzyme Y and Exoenzyme U [8]. ExoS has been proposed as a major virulence factor required for colonization, invasion and bacterial dissemination in burns and chronic pulmonary infections. Also, this exoenzyme is a bifunctional effector protein, with GTPase activating protein (GAP) and ADP-ribosyltransferase (ADPRT) activities which can exert complex effects causing evading the pathogen from the host immune system and cells apoptosis. ExoT is a 53 kDa protein with high homology and enzymatic activity with ExoS. It has been suggested that the GAP activity of ExoS and ExoT could prevent wound healing likely due to the disruption of the actin cytoskeleton, inhibition of bacterial internalization and phagocytosis, and host cells rounding. ExoU possesses a phospholipase A2-like activity resulting in extensive tissue destruction and modulation of the host inflammatory response. This protein contributes greatly in the pathogenesis of highly virulent strains and mostly is associated with the severity of *P. aeruginosa* infections. ExoY with natural adenylate cyclase activity causes an increase in intracellular signal messengers leading to disruption of the actin cytoskeleton and endothelial barriers. The outcome of these events is the dissemination of *P. aeruginosa* into the bloodstream and increased risk of septic shock [7, 8].

Due to the intrinsically and acquired resistance to the broad range of conventional antibiotics, treatment of the infections caused by *P. aeruginosa* has been limited [9]. Recently, the emergence of the multidrug resistance (MDR) *P. aeruginosa* strains producing extended spectrum β-lactamases (ESBLs) and metalo β-lactamases (MBLs) has become a global health concern [10, 11]. It is not surprising that infections caused by MDR strains are associated with significant morbidity and mortality [3, 12]. Therefore, investigation of *P. aeruginosa* virulence genes among MDR strains is waranted to prevent the spread of hyper virulent-resistant strains. Despite the significance of *P. aeruginosa* infection in burn patients, there is currently little local information on the distribution of the virulence factors to estimate the burden of toxin-producing isolates in Iran. The present study aimed to determine the prevalence of virulence factors and antimicrobial resistance profile of *P. aeruginosa* strains isolated from Iranian burn patients.

## Main text

### Methods

#### Study design and bacterial isolates

In this cross-sectional study, a total of 100 clinical isolates of *P. aeruginosa* were obtained from the burn wounds of the patients hospitalized in the Burn Center of Velayat burn injuries hospital (Guilan University of Medical Sciences, Rasht, Iran) during October 2014 to December 2015. This study was performed in accordance with the declaration of Helsinki and approved by the Ethics Committee of Guilan University of Medical Sciences. The exclusion criteria were taking any antibiotic treatment at least 1 week before sample collection. The burn wound samples were taken from the subjects using a sterile swab moistened with sterile physiological saline. Then, the swabs were transferred into tubes containing Stuart’s transport medium (Merck, Germany) within 1 h, and in the laboratory, were transferred onto the brain heart infusion (Merck, Germany) broth and incubated for overnight at 37 °C. All the presumptive isolates on MacConkey agar (Merck, Germany) were identified as *P. aeruginosa* using the standard microbiological methods including Gram staining, capacity for growth at 42 °C, growth on Cetrimide agar (Merck, Germany), oxidase reaction, and IMViC tests. Confirmed *P. aeruginosa* isolates were stored in tryptic soy broth (TSB) (Merck, Germany) containing 30% glycerol at − 80 °C until further study.

#### Antibiotic susceptibility test

The antimicrobial susceptibility test was done by disk diffusion method on Mueller–Hinton agar (Merck, Germany) in accordance with the Clinical and Laboratory Standards Institute (CLSI) recommendations [13]. The following antibiotics were tested; Ceftazidime (30 µg), Piperacillin (100 µg), Gentamicin (10 µg), Tobramycin (10 µg), Amikacin (30 µg), Ciprofloxacin (5 µg), and Imipenem (10 µg) (MAST Co., UK). Each agar plate with 20 ml of Mueller–Hinton was incubated for 16–18 h at 37 °C. Control strains for susceptibility test were *P. aeruginosa* ATCC 27853. MDR was estimated as non-susceptible (including, resistant or intermediate) to at least 1 agent in ≥ 3 antimicrobial categories according to the previously described definitions [14].

#### Genomic DNA extraction and molecular assay

Genomic DNA was extracted from the overnight TSB cultures of *P. aeruginosa* isolates using the boiling method as previously described [15]. The evaluation of *exoT, exoY*, *exoS* and *exoU* genes were done by previously described primers (Table 1) [16]. PCR amplification was performed in 50 µl reaction volume using Taq DNA polymerase. The reaction mixture consisted of 5 µl 1× PCR buffer, 2 µl of each primer, 1 µl MgCl_2_, 0.8 µl each of the dNTPs, 0.6 µl Taq DNA polymerase, and a 2 µl DNA each of isolate. All the reagents were obtained from the Cinnagene Co., Iran. Positive control strain for *exoS*, *exoT*, and *exoY* was *P. aeruginosa* PAO1 and for *exoU* was PA103. The amplicons were resolved in 1.5% agarose gel prepared in 1X TAE (Tris/Acetate/EDTA) buffer and visualized using an ultraviolet light after staining with safe stain loading dye (CinnaGen Co., Tehran, Iran).

**Table 1:** List of used primers in the present study

Primer	Oligonucleotide sequence (5′ to 3′)	Gene	Amplicon size (bp)	Reference
exoT-F	AATCGCCGTCCAACTGCATGCG	*exoT*	152	[16]
exoT-R	TGTTCGCCGAGGTACTGCTC			
exoY-F	CGGATTCTATGGCAGGGAGG	*exoY*	289	
exoY-R	GCCCTTGATGCACTCGACCA			
exoU-F	CCGTTGTGGTGCCGTTGAAG	*exoU*	134	
exoU-R	CCAGATGTTCACCGACTCGC			
exoS-F	GCGAGGTCAGCAGAGTATCG	*exoS*	118	
exoS-R	TTCGGCGTCACTGTGGATGC			

#### Statistical analysis

The analysis was performed by using SPSS™ software, version 21.0 (IBM Corp., USA). The results are presented as descriptive statistics in terms of relative frequency. Values were expressed as the mean ± standard deviation for continuous variables or percentages of the group for categorical variables. Data regarding categorized variables were analysed by Fisher’s exact test and Chi square. A difference was considered statistically significant if the P value was less than 0.05.

### Results

Of totally 100 *P. aeruginosa* isolates included in our study, 65 were recovered from males and 35 from female’s specimens with a mean age of 40.7 ± 23.9 years old, ranging from 1 to 87 year old. Results of the antibiotic susceptibility test revealed that tested *P. aeruginosa* isolates were mostly susceptible to amikacin (61%), ceftazidime (60%), and imipenem (55%). In contrast, the lowest susceptibility rates were seen against tobramycin (32%), ciprofloxacin (38%), and piperacillin (39%). The full results of antibiotic susceptibility profile for *P. aeruginosa* isolates were summarized in Table 2. Moreover, 59 (59%) isolates out of the 100 tested were MDR. The most prevalent MDR pattern were aminoglycosides–penicillins–fluoroquinolones–carbapenems (15%) followed by aminoglycosides–cephems–penicillins–fluoroquinolones–carbapenems (12%), and aminoglycosides–cephems–penicillins–fluoroquinolones (9%).

**Table 2:** The antibiotic susceptibility testing results of 100 *P. aeruginosa* isolates

Class	Antibiotic	Susceptible (%)	Intermediate-resistant (%)	Resistant (%)
Penicillins	Piperacillin	39	8	53
Cephems	Ceftazidime	60	7	33
Carbapenems	Imipenem	55	21	24
Aminoglycosides	Gentamicin	42	4	54
	Amikacin	61	13	26
	Tobramycin	32	7	61
Fluoroquinolones	Ciprofloxacin	38	10	52

The presence of *exoT*, *exoY*, *exoS* and *exoU* genes was detected in 100%, 100%, 59%, and 41% of the tested isolates, respectively. Among the above mentioned genes, the presence of *exoU* gene had a statistically significant association with higher antibiotic resistance, and MDR rate (Table 3).

**Table 3:** Distribution of virulence genes in relation with antibiotic resistance pattern

Class	Antibiotic	*exoS* positive (N = 59)	*exoS* negative (N = 41)	P value	*exoU* positive (N = 41)	*exoU* negative (N = 59)	P value
Penicillins	Piperacillin	29	10	0.013	10	29	0.013
Cephems	Ceftazidime	31	29	0.068	29	31	0.068
Carbapenems	Imipenem	38	17	0.023	17	38	0.023
Aminoglycosides	Gentamicin	32	10	0.003	10	32	0.003
	Amikacin	41	20	0.037	20	41	0.037
	Tobramycin	25	7	0.008	7	25	0.008
Fluoroquinolones	Ciprofloxacin	27	11	0.055	11	27	0.055
MDR positive		30 (50.9)	29 (70.7)	0.047	29 (70.7)	30 (50.9)	0.047

Results presented as No. (%)

### Discussion

Since, the high rate of morbidity and mortality of nosocomial infections in burn patients, the management of these infections is still a great challenge [17]. *P. aeruginosa* as a major cause of burn wound infections exhibits a remarkable ability to acquired resistance to antimicrobial agents [18]. MDR *P. aeruginosa* strains, as a growing public health concern, is moderately resulting from the limited therapeutic options [18].

In the present study, the frequency of MDR in Guilan province (Northern Iran) was estimated 59%, which was lower than that of the studies conducted in capital of Iran in 2011–2013 (93.1%–100%) [19, 20], central parts of Iran in 2015 (95.8%) [21], southwest of Iran in 2014 (76.4%) [22], and Brazil in 2012 (71.4%) [23], whereas it was higher than the studies performed in India in 2014 (33.9%) [24], capital of Iran in 2008 (32.6%) [25], and central parts of Iran in 2013 (26.7%) [26]. These differences in the prevalence of MDR strains might be attributed to the differences in geographical distribution, infection control policies, the nature of infections and sample size.

Carbapenems are important therapeutic agents for the treatment of the infections caused by MDR Gram-negative bacteria, particularly *P. aeruginosa* [27–29]. In our findings, the overall rate of carbapenems-non susceptible isolates was 45%, while this rate in MDR isolates was 67.8%. Such a high rate of carbapenems-resistant isolates in our region can be the result of indiscriminate prescription of these antibiotics as empirical therapies of infections caused by Gram-negative bacteria. However, our results were in consistent with the findings of the previous studies where the increased prevalence of carbapenem-non susceptible isolates in clinical settings was observed [21, 22, 30, 31].

The co-occurrence of *exoT* and *exoY* in our tested isolates was comparable with the previous findings in which the high presence of these genes in clinical isolates of *P. aeruginosa* was shown [32–34], while a minor impact of these exoenzymes on clinical outcome was suggested [34–36]. In the present study, in agreement with the previous reports, the *exoS* gene was the most prevalent exoenzyme in our tested isolates compared with *exoU* [23, 32, 33, 37, 38]. On the other hand, the presence of *exoU* gene compared to *exoS* gene was significantly associated with the higher antibiotic resistance. These findings were in agreement with the results of the studies conducted in Iran and other countries such as Korea, India, and Australia [39–41]. The precise mechanisms by which acquisition of the *exoU* results in higher antibiotic resistance are not known; however, it has been hypothesized that antibiotic-resistance may impose a lower fitness cost in the strains containing these specific genes, consequently leading to better adaptation to the antibiotic-rich clinical environment [40, 42]. The dissemination of MDR *P. aeruginosa* strains with the ability to produce ExoU as a marker for highly virulent strains is a great concern due to restricted therapeutic options to treat these patients.

In summary, our data point out the pattern of MDR and the genetic diversity of type III secretion system associated exoenzymes. Also, the presence of *exoU* gene can be a predictive marker for the persistence of these *P. aeruginosa* strains isolated from the hospitals and consequently a worse clinical condition for the affected patients. However, in view of the limitations of the present study, further studies in larger series would be welcomed.

## Limitations

The present study had some limitations; at first, the gene expression or enzymatic activity of the exoenzymes was not evaluated. Second, due to the lack of a molecular typing method, there was no mention of the coloniality of the isolated strains.

## Abbreviations

T3SS: type III secretion system
Exo: exoenzyme S
MDR: multidrug resistance
MBLs: metalo β-lactamases
TSB: tryptic soy broth
CLSI: Clinical and Laboratory Standards Institute
